# Fatal toxic epidermal necrolysis associated with sinomenine in a patient with primary membranous nephropathy

**DOI:** 10.5414/CNCS111159

**Published:** 2023-08-07

**Authors:** Xue-xia Li, Jun-tao Zhang, Xiao-ying Ding

**Affiliations:** 1 State Key Laboratory of Quality Research in Chinese Medicine, Macao University of Science and Technology, Macao, and; 2 Department of Nephropathy, Zhuhai Hospital of Integrated Chinese and Western Medicine, Zhuhai, Guangdong, China

**Keywords:** toxic epidermal necrolysis, sinomenine, primary membranous nephropathy

## Abstract

Sinomenine (SIN), the alkaloid monomer extracted from *Sinomenium acutum*, is a kind of non-steroidal anti-inflammatory drug widely used in China to treat rheumatoid arthritis (RA) and various glomerular diseases. It has various pharmacological effects such as anti-inflammatory, analgesic, and anti-tumor. As a strong histamine-releasing agent, SIN has drawn increasing attention in regards to its side effects such as allergic, gastrointestinal, and circulatory systemic reactions. In this report, we first described a patient with primary membranous nephropathy (PMN) who was treated with oral intake of SIN and developed medicine-induced toxic epidermal necrolysis (TEN) and subsequently died of septic multi-organ failure. The present case report intends to demonstrate the underestimated side effects of SIN that can eventually lead to death.

## Case presentation 

**Figure 1.**
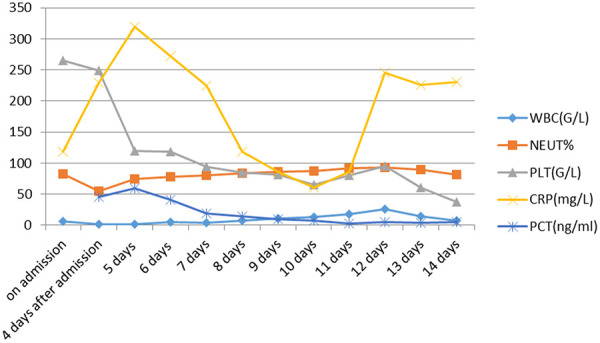
Data for infection.

**Figure 2.**
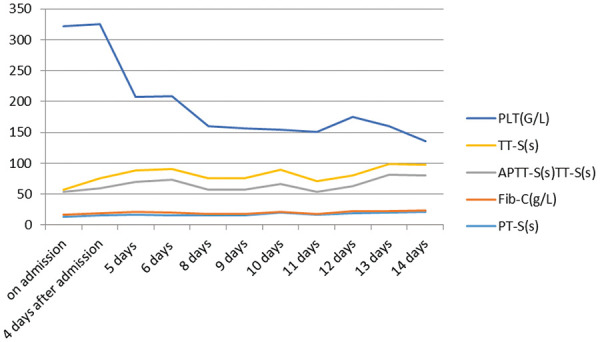
Data for hemorrhage and coagulation.

On November 2, 2020, a 74-year-old man was admitted to our emergency department after presentation with a 2-week history of rashes and itching on the trunk and limbs. Ten months prior to admission, he had been diagnosed with hypertension and membranous nephropathy (PMN) by renal biopsy, accompanied by chronic renal failure with an estimated glomerular filtration rate (eGFR) of 25 mL/min. The patient reported long-term use of benazepril and clopidogrel as well as intermittent use of furosemide. No allergic history was traced. The patient denied a history of smoking or drinking alcohol. Steroids at a dose of 15 mg/d and sinomenine (SIN) at a dose of 120 mg/day were prescribed on September 27 in the outpatient department. Edema was relieved in the first 2 weeks after the prescription. Rash and itching on the trunk and limbs were described on October 17, without coughing or fever. SIN was withdrawn, and loratadine was prescribed. Routine blood test was obtained indicating stable blood cell analysis, liver, and kidney function (Figures 1, 2). The rash and itching was once alleviated but quickly onset after his intake of sea-fish, presented by red and swollen on his face, trunk and limbs, with multiple ulcers of the oral mucosa and pharynx , accompanied with severe pain and poor appetite. 

On admission, physical examination showed a temperature of 36.9 °C, the heart rate was 76 times per minute, the respiratory rate were 20 times per minute, and blood pressure was 153/83 mmHg. Pruritic morbilliform eruption developed on his body and gradually changed into erythroderma. The rash covered over 75% of the body surface, all over his face, chest, abdomen, back, limbs, armpits, perineal area, and mucous. Multiple bullae and ulcers with yellow exudate were present on the lips and oropharyngeal mucosa. Also, there was peripheral edema. Severity-of-illness score for toxic epidermal necrolysis (SCORTEN) on admission was 4 (Table 1). 

**Figure 3.**
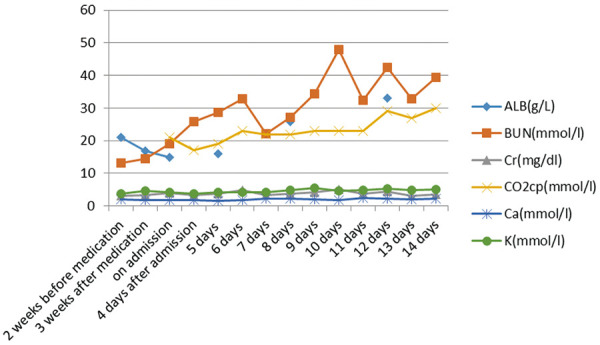
Data for ALB and renal function.

Blood tests for infection, hemorrhage, coagulation as well as heart and renal function were performed (Figures 1, 2, 3). Lymphocytes were assayed by flow cytometry (Table 2). Phospholipase A2 receptor (PLA2R) was positive at a titer of 1/32. Complement C3 and C4 levels, antinuclear antibodies (ANA), anti-double stranded DNA (dsDNA), and anti-neutrophil cytoplasmic antibodies (ANCA) were negative. Liver function was normal. Proteinuria level was 6,264 mg/24h. Electrocardiogram (ECG), chest X ray (Figure 3) and abdominal and urological color ultrasound were normal. 

**Figure 4.**
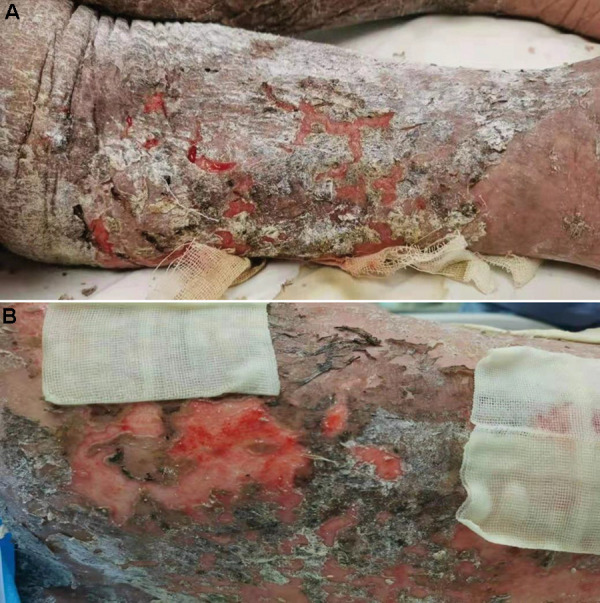
Skin of leg (A) and back (B) showing extensive epidermal denudation with underlying exposed dermis.

**Figure 5.**
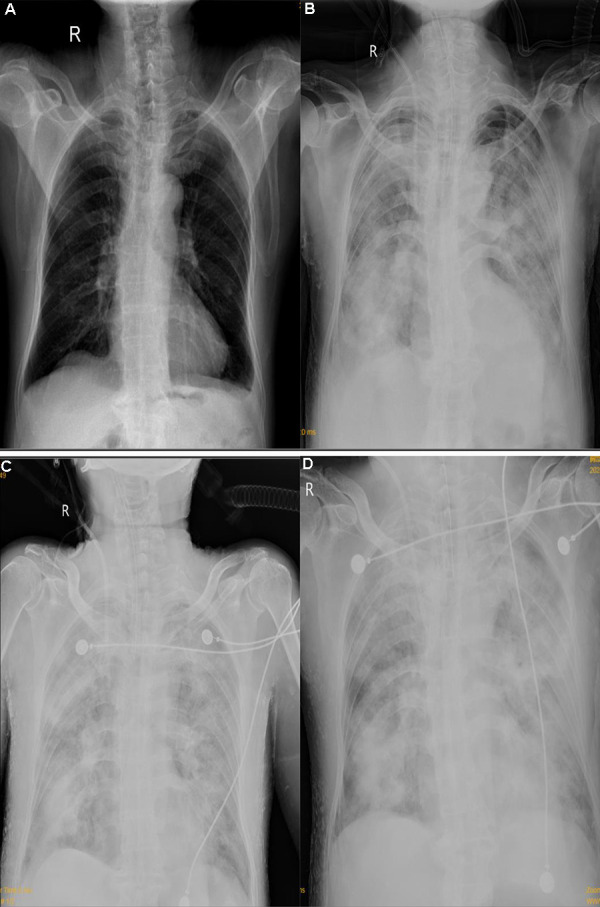
Chest X-ray of the lungs. On admission (A), 12 days after admission (B), 13 days after admission (C), 14 days after admission (D).

The patient was diagnosed with medicine-induced TEN, PMN, CKD, and hypertension. Methylprednisolone at a dose of 80 mg was intravenous injected. Furacilin solution was applied for skin wet packing. The third day after admission, bullae appeared on the abdomen and back, and on the next day, sheets of denuded skin were present on his trunk (Figure 4). At the same time, the patient began to suffer from fever at a temperature of 37.8 °C, and his leukocyte fleetly dropped to 1.7 g/L (Figure 1). The patient was transferred to the intensive care unit (ICU). Specimens from the ulcers and blood were obtained for cultures. Fluids, electrolytes, and albumin were intravenously injected for support. Prednisone and gamma globulin were used intravenously for immuno-regulation. Levofloxacin was applied for eye drops and nitrofural was for skin care, as were sedatives to control pain. Blood perfusion and continuous renal replacement therapy (CRRT) were applied for the onset of worsen kidney and heart function (Figure 3). *Enterococcus faecalis* was cultured from perineal ulcer, and *Morganella morganni spp sibonii* was cultured from other parts of dermal ulcers. *Pseudomonas aeruginosa* was cultured from aerobe and *Staphylococcus aureus* from anaerobe after 4 days of admission. Moxifloxacin was injected for the infection for 24 hours then upgraded to imipenem-cilastatin and teicoplanin. His hemoglobulin reduced rapidly to collapse of coagulation system (Figure 2). There was gastrointestinal bleeding and diffuse alveolar hemorrhage. Erythrocyte and plasma were supported intravenously. Tracheal intubation was performed for ventilatory assistance and bronchofiberscope for alveolar wash. X-ray of the chest rapidly progressed to “great white lungs” in a few days (Figure 5). Both blood and sputum culture yielded *Candida tropicalis*. On the 14^th^ hospital day, hypotension did not respond to medications, probably due to uncontrollable sepsis in his blood and lungs, and the patient died. 

## Discussion 

First described in 1956 [2], and with an incidence of 0.25% in Chinese populations [3], TEN or Lyell’s syndrome is an uncommon idiosyncratic drug reaction involving epidermal necrosis that may be massive, with a mortality rate of 25 – 35% [1]. Antibiotics, anticonvulsant drugs, and non-steroidal anti-inflammatory drugs have been implicated as the most common causative agents [4, 5, 6]. The European Study of Severe Cutaneous Adverse Drug Reactions (EuroSCAR) identified allopurinol as the most common culprit of TEN syndrome [7]. For the first time, we describe SIN as the most possible culprit for TEN. Two weeks after new administration of SIN, our patient suffered from rash and itching which was a delayed anaphylaxis. SIN is a unique plant alkaloid, it potently releases histamine in association with degranulation of tissue mast cells in mammalian tissues, which occurs preferentially in the skin and joint capsules. The released histamine is responsible for the dominant pharmacological actions of SIN, such as vasodilatation, increased vascular permeability, acceleration of the thoracic and peripheral lymph flow, contraction of plain muscles, increased peristalsis of the intestines, and stimulation of gastric acid secretion. Clinical side effects encountered with injected SIN are pruritus in the head and upper parts of the body as well as edema around the lips and eyelids. Oral SIN has been considered to have few side-effects, little knowledge exists about the life-threatening reactions it can induce. 

The pathophysiology of TEN is still not clear. Increasing studies have revealed that HLA alleles are the major genetic determinants of drug hypersensitivity [8]. Certain HLA variants are associated with an increased risk of hypersensitivity reactions to specific drugs. Also, the sensitivity of HLA tests shows a wide range, from 0 to 33% for HLA-B*1502 testing to predict lamotrigine-induced TEN, up to 100% for HLA-B*5701 to predict abacavir hypersensitivity. And HLA-B*5801 testing in Asian populations shows high sensitivity and high specificity for allopurinol-induced TEN: 88 – 100% and 82 – 94%, respectively [9]. HLA-B*1502 carriers were considered to cause allopurinol-related Stevens-Johnson syndrome (SJS)/TEN in Japanese [10]. While in our case, HLA-B*1502 was negative, the drug-gene interaction between SIN and HLA could be further explored. 

In addition, the pathogenesis of TEN is believed to be immune-mediated. Keratinocyte apoptosis followed by necrosis is the pathogenic basis of the widespread epidermal detachment observed in TEN. The etiopathogenesis of it involves the activation of drug-specific T cells [11, 12, 13]. A marked expansion of unique polycytotoxic CD8+ T cell clones play an important role in TEN [13]. Cytotoxic T lymphocytes (CTL) exert drug-specific cytotoxic activity against both autologous B lymphocyte cell lines and keratinocytes, leading to severe and life-threatening TEN, with massive clonal expansion of polycytotoxic skin and blood CD8+ T cells in patients. In our case, B and T lymphocytes remained normal at the very early stage of TEN, with B lymphocytes constantly increasing as TEN developed, and finally polycytotoxic CD8+ T cells were activated. Hardly any report has illustrated how B lymphocytes play a role in medicine-induced TEN, which seems to be activated prior to CD8+ T cell activity. The roles of T lymphocytes and B lymphocytes in TEN caused by SIN needs further exploration. 

There is no specific drug treatments for TEN. In critical cases, anti-sepsis plays an important role in the treatment of it. Sepsis, usually due to *Staphylococcus aureus* or *Pseudomonas aeruginosa* has been reported in 40% of cases with toxic epidermal necrolysis and was the primary cause of death in these patients, as in our case [3]. Intravenous immunoglobulin and corticosteroids might be helpful. Supported care like hemodialysis, hemoperfusion, and respiratory support might also be helpful. In this case, none of the above strategies worked. Monitoring the side-effects of drugs and taking immediate action at the earliest stage of hypersensitivity would have been more effective. 

## Funding 

None. 

## Conflict of interest 

The author declares that the research was conducted in the absence of any commercial or financial relationships that could be construed as a potential conflict of interest. 

**Table 1.** SCORTEN severity-of-illness score for toxic epidermal necrolysis.

Risk factors	0	1	
Age (years)	< 40	≥ 40	1
Associated malignancy	No	Yes	0
Heart rate (beats/min)	< 120	≥ 120	0
Serum urea level (mmol/L)	< 10	≥ 10	1
Percentage of epidermal detachment	< 10%	≥ 10%	0
Serum bicarbonate (mmol/L)	≥ 20	< 20	0
Serum glucose (mmol/L)	< 14	≥ 14	0
SCORTEN			4

The mortality risk depends on the score: SCORTEN 0 – 1 > 3.2%; SCORTEN 2 > 12.1%; SCORTEN 3 > 35.3%; SCORTEN 4 > 58.3%; SCORTEN 5 or more > 90% [1].

**Table 2.** Changes of lymphocytes.

	3 weeks before medication	Admission day	12 days after admission
CD3+ T (50 – 84%)	64	64	40*
CD3+CD4+ T (27 – 51%)	37	33	33
CD3+CD8+ T (15 – 44%)	23	25	6*
CD3+CD4+/CD3+CD8+(0.71 – 2.78)	1.61	1.32	5.99*
NK (7 – 40)			1*
B (5 – 18)		20*	55*

Dynamic changes of the T, B, and NK lymphocytes, with *representing abnormality.
